# Compact Modeling of Two-Dimensional Field-Effect Biosensors

**DOI:** 10.3390/s23041840

**Published:** 2023-02-07

**Authors:** Francisco Pasadas, Tarek El Grour, Enrique G. Marin, Alberto Medina-Rull, Alejandro Toral-Lopez, Juan Cuesta-Lopez, Francisco G. Ruiz, Lassaad El Mir, Andrés Godoy

**Affiliations:** 1 Pervasive Electronics Advanced Research Laboratory (PEARL), Departamento de Electrónica y Tecnología de Computadores, Universidad de Granada, 18071 Granada, Spain; 2 Laboratory of Physics of Materials and Nanomaterials Applied at Environment (LaPhyMNE) LR05ES14, Faculty of Sciences of Gabes, Gabes University, Erriadh City, Zrig, 6072 Gabes, Tunisia

**Keywords:** 2D, biosensor, field-effect transistor, immunosensor, modeling, MoS_2_, sensor, TMD, two-dimensional, Verilog-A

## Abstract

A compact model able to predict the electrical read-out of field-effect biosensors based on two-dimensional (2D) semiconductors is introduced. It comprises the analytical description of the electrostatics including the charge density in the 2D semiconductor, the site-binding modeling of the barrier oxide surface charge, and the Stern layer plus an ion-permeable membrane, all coupled with the carrier transport inside the biosensor and solved by making use of the Donnan potential inside the ion-permeable membrane formed by charged macromolecules. This electrostatics and transport description account for the main surface-related physical and chemical processes that impact the biosensor electrical performance, including the transport along the low-dimensional channel in the diffusive regime, electrolyte screening, and the impact of biological charges. The model is implemented in Verilog-A and can be employed on standard circuit design tools. The theoretical predictions obtained with the model are validated against measurements of a MoS_2_ field-effect biosensor for streptavidin detection showing excellent agreement in all operation regimes and leading the way for the circuit-level simulation of biosensors based on 2D semiconductors.

## 1. Introduction

The rapid detection and continuous monitoring of biological and chemical compounds are of utmost interest for medical purposes, including, e.g., point-of-care solutions, drug detection, genomics, etc. [1,2,3,4,5,6]. The chemical and electrical sensors employed in the detection, conventionally grouped under the common term of biosensors, can be broadly categorized into two classes: label-based sensors and label-free sensors. The former group is characterized by processing the sample (looking for the detection of the target molecules) via measurable parameters, such as fluorescence or colorimetry, employing elaborated procedures and additional equipment that precludes its use for real-time applications. The latter is not conditioned by external facilities or human intervention and enables continuous monitoring and detection. Among them, biosensors based on field-effect transistors (BioFETs) are expected to play a leading role in the field, as they enable the direct integration of the detection of biochemical compounds, the signal acquisition, and the conditioning modules in a single system, reducing the cost, the power consumption, and enabling a portable platform for fast and steady sensing.

In this particular biosensing niche, emerging two-dimensional materials (2DMs)-based field-effect transistors (FETs) constitute an exceptional technological alternative for sensing due to their promising physical and chemical properties. Specifically, their optimum surface-to-volume ratio enhances the electrostatic coupling leading to exceptional sensitivity to the target substances. Additionally, 2D sensors can also be fabricated in a miniaturized size by leveraging the ultimate thickness scalability of 2DMs and show the capability to enhance chemical sensitivity via surface functionalization, inherent flexibility, and great mechanical strength [7,8,9,10,11,12] that redounds in their suitability for the nascent flexible, portable, and/or wearable nanotechnology [13,14]. Furthermore, 2DMs present additional outstanding physical and chemical properties such as amphiphilicity, anisotropic thermal conductivity, anti-reflectance, and corrosion resistance [15]. More importantly, many of these 2DMs, such as graphene and some transition metal dichalcogenides (TMDs), are biocompatible with human body tissues and environmentally friendly [16,17]. In addition to this exceptional collection of properties, 2DM technology is compatible with back-end-of-line processing and thin-film technology [18], enabling the hybrid integration of 2DMs with conventional technologies [19]. These features will allow the development of advanced systems formed by the combination of highly sensitive 2D material-based sensors with signal processing and transmission stages based on mature technologies, which will be of fundamental importance for the deployment of the Internet of Things (IoT) platform. 

However, before the technology readiness level for commercial use of these sensors is reached, many requirements should be considered. Among them, the selectivity issue should be the primary consideration. Ions or molecule selectivity depends on the electrical and chemical properties of the 2DMs and of the ions/molecules themselves. Particularly, 2DMs can interact with target ions/molecules by two distinctive mechanisms: physisorption and chemisorption. Physisorption refers to the interaction between the ions or molecules and the surface of 2DMs without any covalent bonding, while chemisorption takes place when covalent bonds are created between the ions or molecules and the surface of 2DMs. Non-covalent interactions are preferred for biosensing because they result in quick response and fast recovery. The nature of the 2DM-ion/molecule interaction can be adjusted by placing a barrier, in the form of an oxide, between the chemical compounds and the 2DM channel. The presence/absence of the barrier categorize BioFET sensing interfaces into an electrolyte–insulator–semiconductor (EIS) or electrolyte–semiconductor (ES) [20]. The barrier in an EIS BioFET (placed between the electrolyte and the semiconductor) can be functionalized with the aim of attaching receptor agents able to capture the target molecules [2,21] and prevents possible reactions between the ions contained in the solution and the semiconductor surface, i.e., the possible chemisorption, but it reduces the electrical coupling between the device and the molecules [20]. In addition, most of the materials employed as insulator barriers have a hydrophilic nature that hinders their functionalization and reduces the efficiency of the bindings [22]. In contrast, ES BioFETs leave the semiconductor channel in direct contact with the electrolyte, taking advantage of the hydrophobicity of some semiconductors to create an intrinsic barrier; 2DMs such as molybdenum disulfide (MoS_2_), reduced graphene oxide (r-GO), and graphene show the hydrophobic behavior, becoming good candidates to be employed in ES BioFETs [22,23,24].

A wide variety of 2DMs-based BioFETs have already been successfully fabricated and tested, and their operating principles are subject of intense research. Graphene and TMDs, namely MoS_2_ and tungsten diselenide (WSe_2_), lead the race, demonstrating high sensitivity to external stimuli, such as those originating from antigen-antibody binding events [3,4,20,21,25,26,27]. In particular, semiconducting monolayers MoS_2_ and WSe_2_ (with a band gap of about 1.7–1.8 eV [13]) exhibit a reduced leakage current and a high on/off current ratio in FET architectures enabling reliable and accurate biosensing [21,28,29]. In this regard, Sarkar et al. [21] have reported MoS_2_-BioFETs exhibiting the potential to detect streptavidin in concentrations as low as 100 femtomolar (fM), while Wang et al. [2] and Park et al. [4] have also demonstrated that MoS_2_-BioFETs can enable 100–400 fM-level detection limits for the prostate-specific antigen (PSA). Also for the detection of PSA, Hossain et al. [3] have reported an ultra-sensitive WSe_2_-BioFET with a detection limit of 10 fg/mL PSA, the lowest concentration detected so far by any BioFET. Although numerous experimental studies and prototypes have been developed focusing on the characterization of TMD-based immunological FETs (ImmunoFETs) for specific and label-free sensing of proteins through antigen-antibody interaction, there is a noticeable lack of theoretical analysis and models able to rationalize, explain, optimize, and predict the response of the 2D BioFETs. However, in the case of electrolyte-gated graphene-based [30,31,32] and organic (EGOFETs) [33,34,35] field-effect sensors, some works have already been published regarding the modeling of their static and dynamic electrical response, where the latter have recently been also demonstrated as potential candidates for biosensing applications [36,37,38,39]. The performance of BioFETs depends on complex physical and chemical processes taking place at the interface of the solid–liquid materials, including electrolyte screening, site-binding charge, and biomolecule surface binding dynamics [40] that demand a comprehensive theoretical analysis enabling a deeper understanding of the operating principles of 2D BioFETs. To accurately predict the sensor response upon binding of protein analytes, it thus becomes necessary to model these physical and chemical processes [41]. To make progress in this goal, this work proposes a thorough compact model of 2D EIS field-effect biosensors by combining the electrical description of the membrane originating from the eventual formation of a protein layer in the electrolyte [42] and a physics-based model of 2D TMD-based FETs previously developed and tested by some of the authors [43]. The rest of the paper is organized as follows. In Section 2, the theoretical foundations supporting the model are presented. In Section 3, the model for 2D field-effect biosensors is exploited to examine the impact of ionic screening and surface charged groups on the sensor response. The accuracy of the compact model is validated by comparing the simulations against experimental data of an MoS_2_-based streptavidin detector and finally, Section 4 draws the main conclusions.

## 2. Physics-Based Modeling of 2D Field-Effect Biosensors

**Figure 1:**
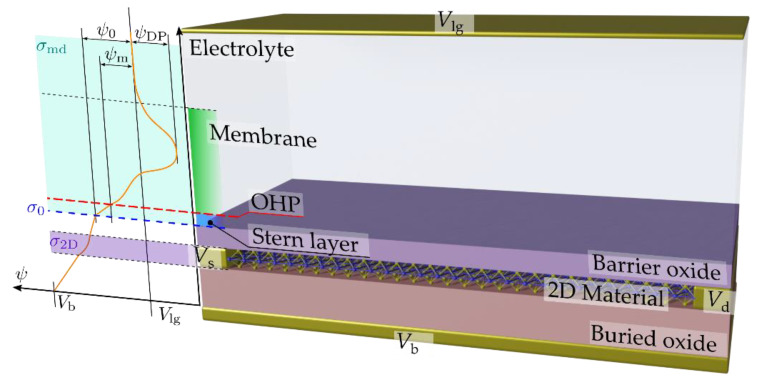
Schematic of a two-dimensional field-effect biosensor. A sketch of the position-dependent potential is also shown, highlighting the surface charge density at the 2D channel ($\sigma_{2D}$), at the oxide-electrolyte interface ($\sigma_{0}$), and at the membrane-diffuse regions of the electrolyte ($\sigma_{\mathrm{md}}$). The latter comprises a charge-free layer (Stern layer) and an ion-permeable membrane due to the presence of charged macromolecules with a diffusion layer located between the barrier oxide surface and the bulk electrolyte. The potential difference from the electrolyte bulk to the barrier oxide surface, $\psi_{0}$, encompasses two contributions originating from a potential drop ($\psi_{0}-\psi_{m}$) across the Stern layer extending between the outer Helmholtz plane (OHP) and the barrier oxide surface, and a potential drop across the ion-permeable membrane layer formed by charged macromolecules and the diffuse layer ($\psi_{m}$).

The sketch of a 2D field-effect biosensor is shown in Figure 1. The device consists of a field-effect transistor where the top-gate metal is substituted by an electrolyte solution with a reference electrode, or liquid gate ($V_{\mathrm{lg}}$), submerged in it. The dielectric layer that covers the 2D channel is functionalized with specific receptors for selectively capturing the target biomolecules. When captured, the charged biomolecules produce a gating effect, which is transduced into an electrical read-out signal in the form of a drain-to-source current or channel conductance change. The inclusion of the insulator layer over the channel acts as a barrier and guarantees an unambiguous field-effect transduction mechanism through the electrostatic control of the channel, i.e., by avoiding eventual chemisorption between the electrolyte and the sensing layer. Additionally, a back-gate electrode, $V_{b}$, located at the bottom of the device provides an extra electrostatic control of the 2D channel through the buried back-gate oxide. 

To achieve a realistic description of the sensor, it is critical to properly model the charge interaction at the 2D channel-insulator and insulator-electrolyte interfaces so as to accurately relate the charge carrier density induced in the sensing layer with the immersed electrode potential. Due to the presence of charged biological macromolecules in the electrolyte, e.g., proteins or single-stranded DNA fragments, an ion-permeable membrane is considered to be placed inside the electrolyte close to the insulator surface (see Figure 1). This layer of biological charged macromolecules is represented as a planar ion-permeable membrane whose potential profile can be described by analytical solutions of the Poisson–Boltzmann equation [42]. 

In the following, this section is split aiming to firstly describe the electrostatics (Section 2.1) and then the carrier transport (Section 2.2) inside a 2D EIS BioFET. 

### 2.1. Electrostatics of a 2D BioFET

The 1D electrostatics through an EIS structure have been previously analyzed by Bousse [44] and extended by Landheer [42] by including the ion-permeable membrane in the electrolyte to describe the effect of attached macromolecules. Following these works, and by applying charge neutrality to the structure, we can get

(1) $$\sigma_{2D}+\sigma_{0}+\sigma_{\mathrm{md}}=0$$

where $\sigma_{2D}$, $\sigma_{0}$, $\sigma_{\mathrm{md}}$ are the surface charge densities associated to the 2D channel (addressed in Section 2.1.1), the oxide-electrolyte interface (Section 2.1.2), and the membrane-diffuse regions of the electrolyte (Section 2.1.3), respectively (see Figure 1). Each of these charges needs to be modeled before charge neutrality can be applied.

#### 2.1.1. Modeling of the Surface Charge Density in a 2D Semiconductor ($\sigma_{2D}$)

The surface charge density in an *n*-type (*p*-type) 2D semiconductor can be calculated by assuming the effective mass approximation, i.e., a parabolic dispersion relationship at the lowest (highest) energies of the conduction (valence) band; and using Fermi–Dirac statistics as follows [43,45]:

(2) σ2D(ϕch)=q{p(ϕch)       if   p-type−n(ϕch)    if   n-type=ϕth{Cdq,plog(1+eϕchϕth)        if   p-type−Cdq,nlog(1+e−ϕchϕth) if   n-type

where q is the elementary charge; $n\left(\phi_{\mathrm{ch}}\right)$ ($p\left(\phi_{\mathrm{ch}}\right)$) is the electron (hole) density; $\phi_{\mathrm{th}}=k_{B}T/q$ is the thermal voltage; $k_{B}$ is the Boltzmann constant; T is the temperature; and $C_{\mathrm{dq},n}=q^{2}D_{0,n}$ ($C_{\mathrm{dq},p}=q^{2}D_{0,p}$) is defined as the electron (hole) degenerated-quantum capacitance that corresponds to the upper-limit achievable when the electron (hole) density becomes heavily degenerated [46]. The conduction (valence) band density of states reads as

(3) $$\{\begin{array}{l} D_{0,p}=g_{h,1}\left(m_{h,1}^{*}/2\pi \hbar^{2}\right)+g_{h,2}\left(m_{h,2}^{*}/2\pi \hbar^{2}\right)\exp\left[-\Delta E_{h,1\to 2}/k_{B}T\right]\;\;\;\mathrm{if}\;\;\;p-\mathrm{type}\\ D_{0,n}=g_{e,1}\left(m_{e,1}^{*}/2\pi \hbar^{2}\right)+g_{e,2}\left(m_{e,2}^{*}/2\pi \hbar^{2}\right)\exp\left[-\Delta E_{e,1\to 2}/k_{B}T\right]\;\;\;\mathrm{if}\;\;\;n-\mathrm{type} \end{array}$$

where ħ is the reduced Planck’s constant; $g_{e,1}$, $g_{e,2}$ ($g_{h,1}$, $g_{h,2}$) are the degeneracy factors; and $m_{e,1}^{*}$, $m_{e,2}^{*}$ ($m_{h,1}^{*}$, $m_{h,2}^{*}$) are the conduction (valence) band effective masses at the first and second lowest (highest) valleys, respectively. The second conduction (valence) valley is non-negligible in most TMDs since the energy separation between the lowest conduction (highest valence) valleys, $\Delta E_{e,1\to 2}$ ($\Delta E_{h,1\to 2}$), is only around 2$k_{B}T$ [47,48]. Thus, two conduction (valence) band valleys may participate in the transport process. On the contrary, the rest of the valleys in the band structure are considered far enough in terms of energy to neglect their contribution to the electrical conduction [49]. This analytical approach has proved its accuracy when modeling MoS_2_-FETs as demonstrated by Cao et al. [50] and Suryavanshi and Pop [51]. The chemical potential, $\phi_{\mathrm{ch}}$, represents the shift of the quasi-Fermi level with respect to the conduction (valence) band edge, and it specifically reads:

(4) $$\phi_{\mathrm{ch}}=\{\begin{array}{c} \left(E_{v}-E_{F}\right)/q\;\;\;\;\;\;\;\;\;\;\;\;\;\;\mathrm{if}\;\;\;\sigma_{2D}>0\;\equiv p-\mathrm{type}\\ \left(E_{c}-E_{F}\right)/q\;\;\;\;\;\;\;\;\;\;\;\;\;\;\;\mathrm{if}\;\;\;\sigma_{2D}<0\;\equiv n-\mathrm{type} \end{array}$$

where $E_{c}$ ($E_{v}$) is the conduction (valence) energy band edge and $E_{F}=\phi /q$ is the quasi-Fermi level that must fulfill the following boundary conditions at the channel edges: $\phi =V_{s}$ at the source and $\phi =V_{d}$ at the drain, where $V_{s}$ and $V_{d}$ are the applied source and drain voltages, respectively.

#### 2.1.2. Modeling of the Charge at the Oxide Surface: Site-Binding Theory ($\sigma_{0}$)

To model the charge at the oxide surface, the site-binding description is assumed to analyze the charge attached to the insulator surface, i.e., the oxide surface sites are considered in equilibrium with the electrolyte by means of a proton exchange [52]. Specifically, the charging/discharging of the surfaces is the result of the uptake or release of protons $H^{+}$ at amphoteric hydroxyl surface groups. The surface reactions to be considered are:

(5) $$-{\mathrm{MOH}}_{2}^{+}↔-\mathrm{MOH}+H^{+}\;-\mathrm{MOH}↔-{\mathrm{MO}}^{-}+H^{+}$$

where −MOH, $-{\mathrm{MO}}^{-}$, and $-{\mathrm{MOH}}_{2}^{+}$ are the neutral, deprotonated, and protonated species, respectively. The dissociation constants are defined as [42]:

(6) $$K_{a}=a_{H^{+}}^{s}\frac{N_{{\mathrm{MO}}^{-}}}{N_{\mathrm{MOH}}}\;\;\;\;\;\;\;\;\;\;K_{b}=a_{H^{+}}^{s}\frac{N_{\mathrm{MOH}}}{N_{{\mathrm{MOH}}_{2}^{+}}}$$

where *N*_i_ (*i* =−MOH, $-{\mathrm{MO}}^{-}$, or $-{\mathrm{MOH}}_{2}^{+}$) represents the corresponding hydroxyl group surface density, and $a_{H^{+}}^{s}$ is the surface proton activity related to the bulk proton activity ($a_{H^{+}}^{b}$) by the Boltzmann distribution as follows:

(7) $$a_{H^{+}}^{s}=a_{H^{+}}^{b}e^{-\frac{\psi_{0}}{\phi_{\mathrm{th}}}}$$

where $\psi_{0}$ is the surface potential drop across the electrolyte-oxide. The total number of surface sites per unit area can be decomposed as:

(8) $$N_{s}=N_{\mathrm{MOH}}+N_{{\mathrm{MO}}^{-}}+N_{{\mathrm{MOH}}_{2}^{+}}$$

and the charge per unit area on the surface is given by:

(9) $$\sigma_{0}=q\left(N_{{\mathrm{MOH}}_{2}^{+}}-N_{{\mathrm{MO}}^{-}}\right)$$

By combining Equations (6)–(9), the surface charge density can be expressed by means of $a_{H^{+}}^{s}$, $N_{s}$, $K_{a}$ and $K_{b}$ as follows:

(10) $$\sigma_{0}=qN_{s}\left(\frac{{\left(a_{H^{+}}^{s}\right)}^{2}-K_{a}K_{b}}{{\left(a_{H^{+}}^{s}\right)}^{2}+K_{b}a_{H^{+}}^{s}+K_{a}K_{b}}\right)$$

#### 2.1.3. Modeling of the Charge Distribution within the Electrolyte: Stern Layer, Ion-Permeable Membrane, and Diffuse Layer ($\sigma_{\mathrm{md}}$)

For the modeling of the electrolyte charge distribution, we assume the formation of an electric double layer (EDL) at the insulator surface with an attached ion-permeable membrane located between its surface and the bulk electrolyte due to the presence of charged macromolecules, e.g., proteins or single-stranded DNA fragments dissolved in the electrolyte that originates a diffuse layer of countercharge. Consequently, within the electrolyte with permittivity $\varepsilon_{w}$ and ion concentration $n_{0}$, two regions are envisaged: (i) a charge-free layer; and (ii) an ion-permeable membrane with a diffusion layer. The outer Helmholtz plane (OHP) is then defined as the plane of the center of the hydrated ions closest to the dielectric in contact with the solution [53]. Thus, in the simplest embodiment of the Gouy–Chapman–Stern model [54], the potential difference from the electrolyte bulk to the oxide surface, $\psi_{0}$, encompasses two contributions originating from the aforementioned regions: (i) a potential drop ($\psi_{0}-\psi_{m}$) across a depleted region of ionic charges close to the surface [54,55,56], namely the Stern layer extending between the OHP and the insulator surface region and characterized by the so-called Stern capacitance ($C_{\mathrm{Stern}}$); and (ii) a potential drop across the ion-permeable membrane layer consisting of the charged macromolecules and a diffuse layer ($\psi_{m}$) [42] (see Figure 1).

Regarding the ion-permeable membrane, the solution concentration within this region is still assumed to be $n_{0}$, although the dielectric constant in the membrane ($\varepsilon_{m}$) can be different from that of the electrolyte due to the presence of macromolecules, the reduced intermolecular hydrogen bonding of the water near the macromolecular surface, and the high counterion concentrations around the molecules [57]. The membrane contains fixed charges with concentration $N_{m}$ uniformly distributed and with a thickness enough to achieve charge neutrality inside it. Therefore, the Donnan potential is reached at the core of the membrane where the electric field is zero. According to Landheer et al. [42], this is a reasonable assumption because charge neutrality in the membrane depends on the screening length inside it. This screening length is shorter than the corresponding one in the electrolyte because the fixed charge in the membrane attracts ions, mostly when $N_{m}\gg n_{0}$ (low solution concentration). Beyond the membrane layer, the diffuse layer is originating from the presence of mobile hydrated ions in the electrolyte. This approach is also adopted by Fernandes et al. in [58], where the Donnan potential is expressed as [42]:

(11) $$\psi_{\mathrm{DP}}=\phi_{\mathrm{th}}\sinh^{-1}\left(\frac{N_{m}}{2n_{0}}\right)=\phi_{\mathrm{th}}\log\left(\frac{N_{m}}{2n_{0}}+\sqrt{1+{\left(\frac{N_{m}}{2n_{0}}\right)}^{2}}\right)$$

As $\psi_{\mathrm{DP}}$ is considered to drop across the membrane, the total net charge density in the membrane and diffuse layer in the electrolyte reads as:

(12) $$\sigma_{\mathrm{md}}=-\mathrm{sgn}\left(\psi_{m}-\psi_{\mathrm{DP}}\right)2\sqrt{qn_{0}\phi_{\mathrm{th}}\varepsilon_{m}}{\left(\cosh\left(\frac{\psi_{m}}{\phi_{\mathrm{th}}}\right)-\cosh\left(\frac{\psi_{\mathrm{DP}}}{\phi_{\mathrm{th}}}\right)-\left(\frac{\psi_{m}-\psi_{\mathrm{DP}}}{\phi_{\mathrm{th}}}\right)\sinh\left(\frac{\psi_{\mathrm{DP}}}{\phi_{\mathrm{th}}}\right)\right)}^{1/2}$$

Following the approach proposed by Fernandes et al. in [58], Dak et al. in [59], and validated by Martinoia and Massobrio in [60], $\sigma_{2D}$ is considered negligible compared with the rest of charge densities in Equation (1). Under this consideration, we obtain the following system of coupled equations

(13) $$\sigma_{0}=-\sigma_{\mathrm{md}}\to \{\begin{array}{c} \psi_{0}=\psi_{m}+\frac{\sigma_{0}\left(\psi_{0}\right)}{C_{\mathrm{Stern}}}\\ \psi_{m}=\psi_{0}+\frac{\sigma_{\mathrm{md}}\left(\psi_{m}\right)}{C_{\mathrm{Stern}}} \end{array}$$

**Figure 2:**
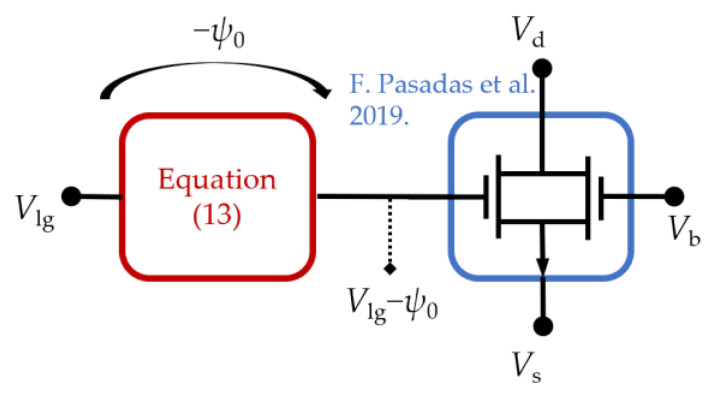
Equivalent circuit of the Verilog-A compact model presented in this work. The pH- and analyte-dependent potential drop in the electrolyte represented by the red box is decoupled from the surface charge density at the 2D channel and calculated by solving Equation (13). The blue box represents the large-signal model for 2D semiconductor-based field-effect transistors developed by the authors in [43].

From the perspective of a circuit-level model, the system in Equation (13) can be solved as a sub-circuit block that plays the role of a non-linear voltage source $-\psi_{0}$ that accounts for the voltage drop across the electrolyte and depends on the pH or analyte concentration [58,59,60]. To solve the system of equations in Equation (13), a construct is implemented in Verilog-A to force the circuit simulator to obtain $\psi_{0}$ during run-time [43,61,62]. The resulting equivalent circuit for 2D BioFETs is shown in Figure 2. Thanks to this approach, the potential drop in the electrolyte $-\psi_{0}$ is decoupled from the surface charge density at the sheet channel, allowing the calculation of the charge density in the 2D channel of a double-gated EIS BioFET as follows:

(14) $$\sigma_{2D}=-C_{\mathrm{tox}}\left(V_{\mathrm{lg}}-V_{\mathrm{go}}-\psi_{0}-\phi +\phi_{\mathrm{ch}}\right)-C_{\mathrm{box}}\left(V_{b}-V_{\mathrm{bo}}-\phi +\phi_{\mathrm{ch}}\right)$$

where $C_{\mathrm{tox}}=\varepsilon_{\mathrm{tox}}/t_{\mathrm{tox}}$ ($C_{\mathrm{box}}=\varepsilon_{\mathrm{box}}/t_{\mathrm{box}}$) is the top- (back)-oxide capacitance per unit area, with $\varepsilon_{\mathrm{tox}}$ ($\varepsilon_{\mathrm{box}}$) the top- (back)-dielectric permittivity and $t_{\mathrm{tox}}$ ($t_{\mathrm{box}}$) the top- (back)-oxide thickness. The liquid- (back)-gate offset voltage $V_{g0}$ ($V_{b0}$) comprises the work function difference between the electrode and the 2D channel as well as the additional fixed charge owing to impurities or doping [63].

As depicted in Figure 2, the large-signal model can be conceptually split into two parts. One, represented by the blue box, is able to predict the electrical read-out of an electrostatically gated 2D channel as far as the band structure of the semiconductor can be modeled under an effective mass approach, i.e., the dispersion relationship at the conduction/valence band minima/maxima can be approximated by a parabola [43,51]. On the other hand, the Verilog-A construct, represented by the red box in Figure 2, determines the voltage drop at the electrolyte generated by charged macromolecules due to the targets and receptors if they can be described by an ion-permeable membrane. In this regard, the model can be generalized not only to a broader class of 2D semiconductors but also to different substances/analytes [5], such as, e.g., the biotin and streptavidin pair, or avidin, which also shows affinity for biotin [64]. The model would also be able to predict the sensitivity in response to DNA hybridization, where the ensemble of surface-bound oligomers can be described as a charged ion-permeable membrane [42,65]. It is worth noting that the model assumes the a priori functionalization of the dielectric layer with specific receptors for selectively capturing the target biomolecules. From the perspective of theoretical modeling, the analysis of the specificity is not trivial and compact models have to rely on previous biochemical research on the interactions of the receptor with other molecules, with the receptor-target complex being eventually described in terms of its charge.

### 2.2. Drain Current Transport through a 2D EIS BioFET

In the diffusive regime, the drain current of a 2D EIS BioFET can be accurately calculated by the following closed-form expression [45]:

(15) $$I_{\mathrm{ds}}=\{\begin{array}{c} \mu_{p}\frac{W}{L}C_{\mathrm{dq},p}\phi_{\mathrm{th}}^{2}\left[\left(1+\frac{C_{\mathrm{dq},p}}{C_{\mathrm{tb}}}\right)\left(\frac{u_{d}^{2}-u_{s}^{2}}{2}\right)+\left(e^{-u_{s}}-e^{-u_{d}}\right)\right]\;\;\;\;\;\mathrm{if}\;\;\;p-\mathrm{type}\\ \mu_{n}\frac{W}{L}C_{\mathrm{dq},n}\phi_{\mathrm{th}}^{2}\left[\left(1+\frac{C_{\mathrm{dq},n}}{C_{\mathrm{tb}}}\right)\left(\frac{u_{s}^{2}-u_{d}^{2}}{2}\right)+\left(e^{-u_{d}}-e^{-u_{s}}\right)\right]\;\;\;\;\;\mathrm{if}\;\;\;n-\mathrm{type} \end{array}$$

where W and L are the channel width and length, respectively; $\mu_{n}$ ($\mu_{p}$) is the electron (hole) effective mobility; $C_{\mathrm{tb}}=C_{\mathrm{tox}}+C_{\mathrm{box}}$ is the sum of the geometrical top- and back-oxide capacitances per unit area; and the function $u\left(\phi_{\mathrm{ch}}\right)$ is defined as follows:

(16) u(ϕch)={log(1+eϕchϕth)        if   p-typelog(1+e−ϕchϕth)        if   n-type

where $u_{s}=u\left(\phi_{\mathrm{ch},s}\right)$ and $u_{d}=u\left(\phi_{\mathrm{ch},d}\right)$ are calculated from the electrostatics in Equations (2) and (14) as $\phi_{\mathrm{ch},s}={\phi_{\mathrm{ch}}|}_{\phi =V_{s}}$ and $\phi_{\mathrm{ch},d}={\phi_{\mathrm{ch}}|}_{\phi =V_{d}}$.

## 3. Results and Discussion

In this section, we provide a validation of the theoretical modeling of the EIS 2D BioFETs addressed in Section 2. To do so, we compare the outcome of the compact model against experimental measurements of a MoS_2_-FET performing as both a pH detector (Section 3.1) and a streptavidin biosensor (Section 3.2). The details of the device fabrication and characterization are reported by Sarkar et al. in [21]. The MoS_2_ channel was obtained by micromechanical exfoliation technique and was transferred onto 270 nm SiO_2_/highly doped Si substrate. The source/drain metal contact stacks are 60 nm/100 nm Ti/Au and, importantly, are passivated with a dielectric layer to protect them from the electrolyte, thus, preventing eventual direct contact between the electrolyte and the edge contacts that would allow the direct adsorption of ions and/or biomolecules. The role of the oxide barrier is played by a 30 nm thick layer of HfO_2_ [21]. An Ag/AgCl reference electrode is used to bias the electrolyte solution formed by 0.01 × Phosphate Buffered Saline (PBS). Table 1 summarizes the parameters and values characterizing the device, electrolyte, ion-permeable membrane, and electrolyte-oxide interface used in the simulation. 

### 3.1. MoS_2_-FET as an Ion-Sensitive Sensor

**Figure 3:**
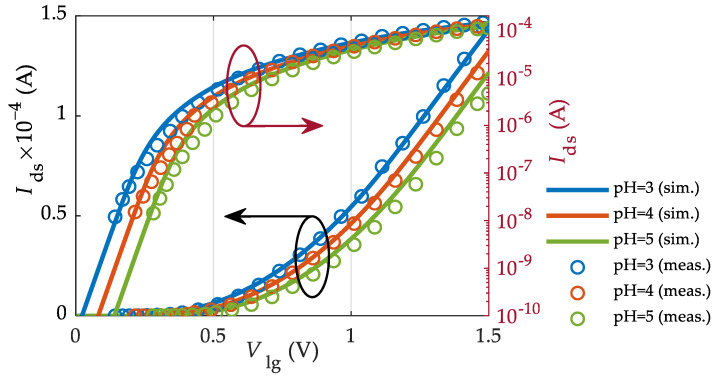
Simulation (solid lines) and measurements (symbols) of the drain current of the MoS_2_-FET described in Table 1 and reported in [21] for different pH = 3 (blue), 4 (red), and 5 (green). Left (right) axis corresponds to a linear (logarithmic) scale.

The operation of the MoS_2_-FET is first analyzed when the pH of the electrolytic solution is modified. Figure 3 shows the simulated and measured transfer characteristics ($I_{\mathrm{ds}}-V_{\mathrm{lg}}$ curves) of the *n*-type MoS_2_-based ion-sensitive FET for three different pH values (pH = 3, 4, 5) in linear (left) and logarithmic (right) scales. To perform the simulation of the device as an ion-detector, the charge concentration in the membrane is set to $N_{m}=0$ (which implies that $\psi_{\mathrm{DP}}=0$) and the permittivity of the membrane is assumed to be the same as the electrolyte, namely $\varepsilon_{m}\to \varepsilon_{w}=80\varepsilon_{0}$.

Indeed, we check that $N_{m}=0$ gets back to our previously reported compact model for 2D EIS ion-sensitive FETs (ISFETs) reported in [71]. The agreement between the model outcome and measurements is excellent for all regimes of operation as shown in Figure 3. The performance of this MoS_2_ technology for ion detection in terms of current and voltage sensitivities for the different operation regions can also be found in [71].

### 3.2. MoS_2_-FET as a Biosensor of Charged Macromolecules

The specific capability of the MoS_2_-BioFET to detect biomolecules is tested on the well-known biotin—streptavidin interaction [72], where the biotin and streptavidin pair act as models for receptor and target molecules, respectively.

**Figure 4:**
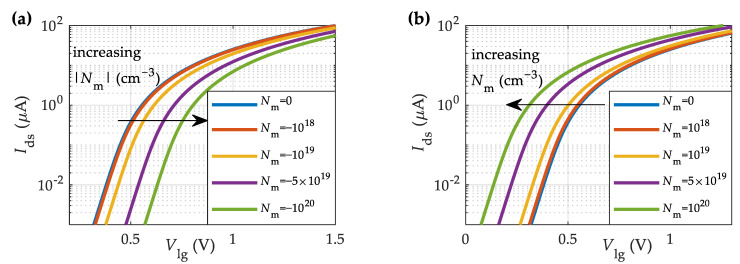
Simulated transfer characteristics of the MoS_2_-BioFET described in Table 1 for different (**a**) negative, and (**b**) positive membrane charge densities (*N_s_* = 0 cm^−2^ and *i*_0_ = 1 mM).

First, we explore the impact on the device characteristics of the charge concentration inside the membrane formed by the macromolecules. In this regard, Figure 4a,b shows the transfer characteristics for the MoS_2_-BioFET described in Table 1 at different negative and positive charge concentrations in the membrane, respectively. To boost the sensitivity, the number of surface sites and the salt concentration has been set to $N_{s}=0$ cm^−2^ and $i_{0}=1$ mM, respectively. An increase in the negative (positive) membrane charge density, $N_{m}$, shifts the curves towards higher (lower) $V_{\mathrm{lg}}$ values, thus increasing (reducing) the threshold voltage, as shown in Figure 4a,b.

**Figure 5:**
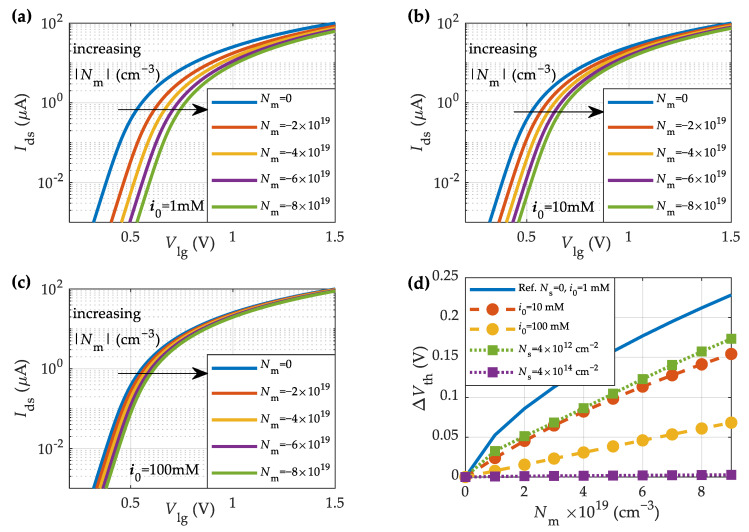
Impact of the varying salt concentration on the sensing response of the MoS_2_-BioFET described in Table 1. The ionic concentration ($i_{0}$) is (**a**) 1 mM; (**b**) 10 mM; and (**c**) 100 mM. (**d**) Threshold voltage shift versus the charge concentration in the membrane ($N_{m}$) for different salt concentrations ($i_{0}$) and binding surface sites ($N_{s}$). The solid blue line is the reference scenario with $N_{s}=0$ cm^−2^ and $i_{0}=1$ mM.

Next, we examine the impact of ionic screening on the device sensitivity for three different salt concentrations, namely, 1 mM, 10 mM, and 100 mM, as shown in Figure 5a–c, respectively. The lower the salt concentration, the higher the sensitivity to variations in the membrane charge density. This behavior can be explained by analyzing the shift of the threshold voltage ($\Delta V_{\mathrm{th}}$) with the membrane charge density, depicted in Figure 5d. The increase in the salt concentration from 1 mM to 100 mM causes a considerable reduction in the voltage sensitivity. If the oxide barrier surface is highly charged (due to a noticeable density of hydroxyl groups on the HfO_2_ surface), the variations in $V_{\mathrm{th}}$ would also be significantly mitigated.

To capture the impact of the streptavidin concentration on the MoS_2_-BioFET electrical characteristic, it is essential to describe the membrane concentration of biotin–streptavidin macromolecules ($N_{m}$) with respect to the density of receptors on the sensor surface (biotin, $N_{r}$), the concentration of targets (streptavidin, $N_{t}$), the binding constant ($K_{c}$), the macromolecule height ($h_{m}$), and the number of fundamental charges per molecules ($k_{q}$). The basic Langmuir adsorption approach is assumed for modeling the adsorption of species onto a surface [73] resulting in:

(17) $$N_{m}=\frac{k_{q}}{h_{m}}N_{r}\left(\frac{K_{c}N_{t}}{1+K_{c}N_{t}}\right)$$

The volumetric density of fixed charged macromolecules in the membrane is calculated according to its length, the surface density of targets and receptors and the number of charges per molecule. The biotin–streptavidin system is attached by one of the strongest non-covalent protein—ligand interactions, with a maximum binding constant of 10^15^ M^−1^ and a default value of $K_{c}$ = 10^13^ M^−1^ [69]. After functionalization, the hydroxyl group surface density is assumed to be reduced to 1% of the available sites on the HfO_2_ surface ($N_{s}$ = 4 × 10^12^ cm^−2^) with a biotin surface density of $N_{r}$ = 2.3 × 10^13^ cm^−2^. While the biotin molecule is neutral [72], the streptavidin molecule is negatively (positively) charged in the solution when the pH is higher (lower) than the isoelectric point, pI, namely, pH > pI (pH < pI) [64]. Therefore, depending on the pH value, the expected impact of the charged molecules will be either the one shown in Figure 4a (pH > pI) or Figure 4b (pH < pI).

**Figure 6:**
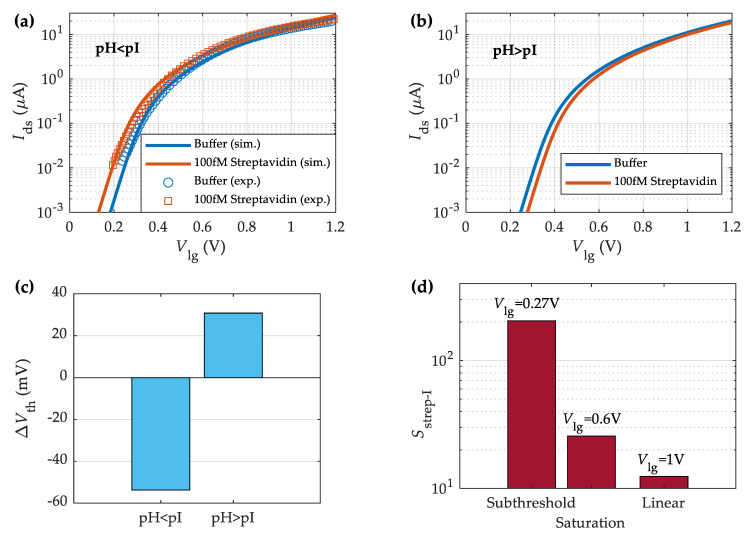
Measured [21] (symbols) and simulated (solid lines) streptavidin sensing response of the MoS_2_-BioFET described in Table 1 under different pH solutions, specifically, (**a**) acidic solution (pH = 3) and (**b**) basic solution (pH = 9). (**c**) Change in threshold voltage with streptavidin (in acidic and basic solutions). (**d**) Streptavidin current sensitivity of the MoS_2_-BioFET in the different transistor operation regions at pH = 3.

Figure 6a–b shows streptavidin sensing results of a MoS_2_-BioFET in two different pH environments, namely, an acidic (pH = 3) and a basic (pH = 9) solution. The corresponding numbers of charges per molecule are $k_{q}=$ +22.3 and −15.2 for pH = 3 and pH = 9, respectively, as obtained from the PROPKA algorithm which predicts the values of ionizable groups in proteins and protein-ligand complexes [64,74,75]. Table 1 collects a summary of the parameters characterizing the electrolyte, membrane, and electrolyte-oxide interface. The point of charge neutrality, pI, of streptavidin molecules is ~5.04, thus, the protein will be positively charged in the acidic solution at pH = 3, while negatively charged in the basic solution at pH = 9. Specifically, in the former (Figure 6a), the drain current increases with the addition of streptavidin for a fixed $V_{\mathrm{lg}}$, i.e., $V_{\mathrm{th}}$ is negatively shifted as addressed in Figure 6c. In contrast, the current is reduced in the basic solution (pH = 9) in the presence of the target molecule, streptavidin, namely, $V_{\mathrm{th}}$ is positively shifted (Figure 6c). This behavior agrees with the results shown in Figure 4a–b and with other previously reported biotin–streptavidin field-effect-based sensing experiments [21,76]. Hence, the good agreement depicted between the proposed model and the experimental data demonstrates its capability to predict the biosensor response to the presence streptavidin diluted in solutions of variable pH. 

**Figure 7:**
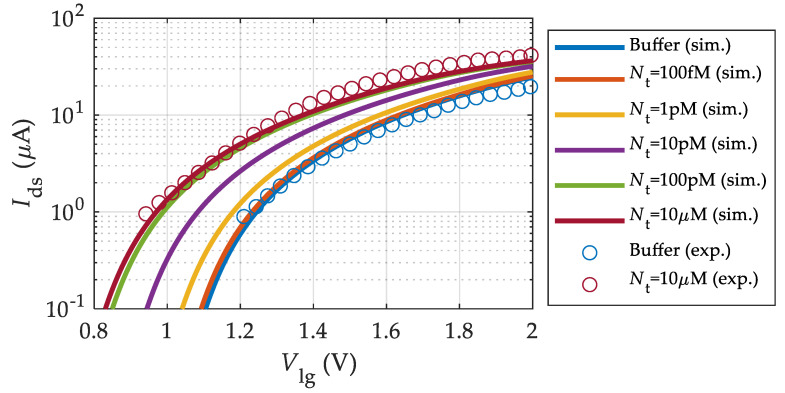
Transfer characteristics of the MoS_2_-BioFET under various streptavidin concentrations ($N_{t}$) at pH = 4.75. Symbols represent the measurements [21] and solid lines depict the model outcome.

A key figure of merit for biosensors is the current sensitivity, $S_{\mathrm{strep}-I}$, defined as the relative change of the BioFET current before and after the streptavidin binding divided by the lowest of both currents [21]. Figure 6d shows the comparison of $S_{\mathrm{strep}-I}$ in the different transistor operating regions; namely, subthreshold, saturation, and linear regions. $S_{\mathrm{strep}-I}$ reaches a value of about 200 in the subthreshold region for a streptavidin solution of $N_{t}=$100 fM. This value is very close to that obtained directly from the measured transfer characteristics (196 in the subthreshold region [21]). For the saturation and linear regions, the sensitivity is notably reduced. Finally, we investigate the biosensor response to different streptavidin concentrations, $N_{t}$. Figure 7 shows the simulated transfer characteristics of the MoS_2_-BioFET described in Table 1 when streptavidin solutions with concentrations ranging from 100 fM to 10 µM are employed. The experimental measurements for the buffer and $N_{t}$ = 10 µM [21] are also included in Figure 7 (symbols), showing a good fit between them and a clearly monotonic behavior of $I_{\mathrm{ds}}$ with $N_{t}$. The net charge associated to the streptavidin is positive for the considered pH = 4.75, and thus, the drain current increases due to the reduction in the threshold voltage with the increase in streptavidin concentration.

## 4. Conclusions

We have developed a comprehensive compact model able to describe the electrical response of two-dimensional electrolyte-insulator-semiconductor field-effect biosensors where the macromolecules formed by receptors and targets attached to the insulator surface are represented by a charged ion-permeable membrane. The model accounts for the surface-related physical and chemical processes that cause a significant influence on the biosensor performance, including the electrolyte screening, site-binding charge, and biomolecule charges at the electrolyte side, and combines them with a drift-diffusion description of the electron transport in the semiconductor channel. The model has been implemented in Verilog-A and, therefore, it is compatible with standard commercial circuit simulators. The theoretical predictions have been validated against the electrical response of an experimental MoS_2_-biosensor in the presence of variable pH and streptavidin concentrations showing excellent agreement in all these situations. The proposed model enables a straightforward application for the biosensing of different macromolecules, making use of a variety of 2D semiconductors and novel structures, and enabling its analysis and integration at the circuit level.

## Figures and Tables

**Table 1:** Parameters of the modeled MoS_2_-BioFET.

Parameter	Value	Parameter	Value
L (µm)	5 [21]	$\mu_{n}$ (cm^2^/Vs)	20
W (µm)	20 [21]	$\varepsilon_{w}$	80ε_0_
$t_{\mathrm{tox}}$ (nm)	30 [21]	$\varepsilon_{m}$	80ε_0_
$\varepsilon_{\mathrm{tox}}$ [HfO_2_]	25	${\mathrm{pK}}_{a}$	7 [66]
$t_{\mathrm{box}}$ (nm)	270 [21]	${\mathrm{pK}}_{b}$ _b_	7 [66]
$\varepsilon_{\mathrm{box}}$ [SiO_2_]	3.9	$C_{\mathrm{Stern}}$ (μF/cm^2^)	20 [67]
$V_{\mathrm{go}}$ (V)	0.48	$i_{0}$ (mM)	1
$V_{\mathrm{bo}}$ (V)	0	$N_{s}$ (cm^−2^)	4 × 10^14^ [66]
$m_{e,1}^{*}/m_{0}$	0.54 [68]	$N_{r}$ (cm^−2^)	2.3 × 10^13^
$m_{e,2}^{*}/m_{0}$	0.58 [68]	$N_{t}$ (fM)	100
$g_{e,1}$	2	$K_{c}$ (M^−1^)	10^13^ [69]
$g_{e,2}$	6	$h_{m}$ (nm)	5 [70]
$\Delta E_{e,1\to 2}$ (eV)	0.07 [47]	$V_{\mathrm{ds}}$ (V)	1

The pH at the point of zero charge is defined as ${\mathrm{pH}}_{\mathrm{pzc}}=\left({\mathrm{pK}}_{a}+{\mathrm{pK}}_{b}\right)/2$, with ${\mathrm{pK}}_{a}=-{log}_{10}\left(K_{a}\right)$, ${\mathrm{pK}}_{b}=-{log}_{10}\left(K_{b}\right)$, and $\mathrm{pH}=-{log}_{10}\left(a_{H^{+}}^{b}\right)$. The salt concentration can be calculated as $n_{0}=N_{A}i_{0}$, where $N_{A}$ is the Avogadro constant and $i_{0}$ is the ionic molar concentration of the solution. The parameters $N_{s}$, $N_{r}$, $N_{t}$, and $i_{0}$, will be modified for the different experiments.

## Data Availability

The Verilog-A model for 2D EIS BioFETs is available from the corresponding author (fpasadas@ugr.es) upon reasonable request.
